# Decoding the neurotoxic effects of propofol: insights into the RARα-Snhg1-Bdnf regulatory cascade

**DOI:** 10.1152/ajpcell.00547.2023

**Published:** 2024-04-15

**Authors:** Yuhai Xu, Xin Xin, Tianzhu Tao

**Affiliations:** Department of Anesthesiology, Air Force Medical Center, Beijing, People’s Republic of China

**Keywords:** Bdnf, propofol, RARα, RARa, neurotoxicity, Snhg1

## Abstract

The potential neurotoxic effects of propofol, an extensively utilized anesthetic, underline the urgency to comprehend its influence on neuronal health. Insights into the role of the retinoic acid receptor-α, small nucleolar RNA host gene 1, and brain-derived neurotrophic factor (RARα-Snhg1-Bdnf) network can offer significant advancements in minimizing these effects. The study targets the exploration of the RARα and Snhg1 regulatory network’s influence on Bdnf expression in the realm of propofol-induced neurotoxicity. Harnessing the Gene Expression Omnibus (GEO) database and utilizing JASPAR and RNA-Protein Interaction Prediction (RPISeq) database for projections, the study embarks on an in-depth analysis employing both in vitro and in vivo models. The findings draw a clear link between propofol-induced neurotoxicity and the amplification of RAR signaling pathways, impacting hippocampal development and apoptosis and leading to increased RARα and Snhg1 and decreased Bdnf. Propofol is inferred to accentuate neurotoxicity by heightening RARα and Snhg1 interactions, culminating in Bdnf suppression.

**NEW & NOTEWORTHY** This study aimed to decode propofol’s neurotoxic effects on the regulatory cascade, provide insights into the RARα-Snhg1-Bdnf interaction, apply extensive validation techniques, provide a detailed analysis and exploration of propofol’s neurotoxicity, and offer a comprehensive approach to understanding molecular interactions.

## Graphical abstract

**Figure:**
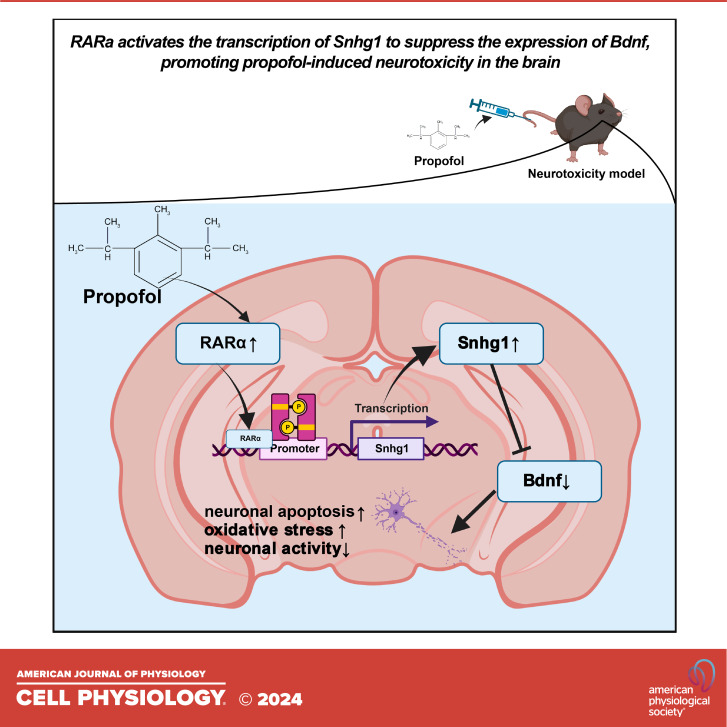

## INTRODUCTION

Propofol is a commonly employed medication for anesthesia and pain control. However, recent studies have indicated that it may induce neurotoxicity in the brain (1). This discovery has significantly impacted the medical community because it has prompted a thorough examination of the challenges associated with propofol use in clinical applications and the variations in patient outcomes (2). At present, no practical method has been discovered to offset the neurotoxicity caused by propofol. As a result, doctors must exercise extreme caution when administering this medication to minimize potential patient risks (3).

Brain-derived neurotrophic factor (BDNF) is a neurotrophic growth factor found in the brain. It plays a crucial role in promoting neurons’ survival, development, and maintenance and also exhibits a protective effect on them (4). In several neurodegenerative diseases, such as Alzheimer’s disease and Parkinson’s disease, a decrease in BDNF expression is commonly linked to the initiation and advancement of the diseases (5–7). Nevertheless, the involvement of BDNF in the neurotoxic effects of propofol has not been thoroughly clarified (8). Retinoic acid receptor-α (RARα) plays a crucial role in the retinoic acid (RA) signaling pathway, regulating various essential physiological processes, including cell growth and differentiation (9). The long noncoding RNA (lncRNA) small nucleolar RNA host gene 1 (Snhg1) has emerged as a prominent subject of investigation in recent years. It has been determined to be pivotal in various diseases, notably neurological disorders (10, 11). However, it remains uncertain whether RARα and Snhg1 contribute to the neurotoxicity induced by propofol in the brain and the relationship between these factors and BDNF (12). Significant strides have been made in recent years in studying the essential genes and transcription factors associated with brain neurotoxicity targets. Of particular focus are RARα, Snhg1, and Bdnf (13). RARα, also called retinoic acid receptor-α, plays a critical role in the normal functioning of neurodevelopment and the nervous system (14). Snhg1 is a noncoding RNA that modulates gene expression in neurons, whereas Bdnf plays a crucial role in the maintenance and development of nerve cells (15). A thorough understanding of the functions and interrelationships of these targets holds promise for providing new opportunities in treating neurological disorders (16).

This study aims to investigate the potential mechanisms underlying propofol-induced neurotoxicity in the brain. The significance of this discovery is that propofol may hinder the expression of the Bdnf gene by enhancing the binding between RARα and the Snhg1 promoter region. This finding offers compelling evidence for the investigation of additional neuroprotective techniques and can potentially enhance propofol’s safety in clinical settings.

## MATERIALS AND METHODS

### Propofol Target Gene Screening

Propofol’s two- and three-dimensional (2-D and 3-D) chemical structures (Compound CID: 4943) were obtained from the PubChem database (https://pubchem.ncbi.nlm.nih.gov/). The 3-D chemical structure was saved in the “SDF” format and uploaded to the PharmMapper server (http://lilab-ecust.cn/pharmmapper/). The reverse docking server PharmMapper was used to simulate the molecular-protein docking and identify potential targets for propofol. The target range was set to “Human Protein Targets Only,” while the remaining settings were defaulted (17).

### Differential Analysis of High-Throughput Transcriptome Sequencing Datasets

The gene expression profile datasets GSE60820, GSE61616, and GSE212732 were obtained from the Gene Expression Omnibus (GEO) database (https://www.ncbi.nlm.nih.gov/gds). The GSE60820 dataset includes samples of cortical tissue from five mice in the sham surgery group and five mice with ischemic brain injury. The GSE61616 dataset contains samples of cortical tissue from five rats in the sham surgery group and five rats with ischemic brain injury. The GSE212732 dataset consists of samples of cortical tissue from 4 rats in the sham surgery group and 11 rats with ischemic brain injury. Differential analysis was performed using the “limma” package in the R programming language, with a significance level set at *P* < 0.05 to select differentially expressed genes. We identified differentially expressed lncRNAs based on the GSE60820 dataset and differentially expressed mRNAs based on the GSE60820, GSE61616, and GSE212732 datasets (18).

### Propofol Target Transcription Factor Screening

The transcription factor dataset was obtained through the hTFtarget database (http://bioinfo.life.hust.edu.cn/hTFtarget#!/tf). Next, we used the Venn diagram tool on the online analysis website, sanger-box (http://vip.sangerbox.com/home.html), to intersect the target data for regulating propofol-induced neurotoxicity in the brain and the transcription factor dataset. It allowed us to obtain potential differentially expressed transcription factors associated with propofol-induced neurotoxicity in the brain (18).

### Functional Enrichment Analysis of Candidate Targets

The functional clustering of candidate targets was performed using the bioinformatics tools available on Xiantao Scholar (https://www.xiantao.love/products/apply/). Gene Ontology (GO) and Kyoto Encyclopedia of Genes and Genomes (KEGG) analyses were conducted to explore biological processes (BP), molecular functions (MF), and KEGG pathways. The screening criteria were set at a significance level of *P* < 0.05, and the enrichment analysis results based on *P* values were visualized using circular plots (19).

### Screening of lncRNAs and Prediction of Their Binding Transcription Factors

To extract features of lncRNA, a multivariate Cox study utilizing Lasso regression was conducted using the “glmnet” package in R language. High expression differentials in lncRNA were chosen from the pool of candidate lncRNA as critical lncRNA. Subsequently, these key lncRNA promoter sequences were obtained from the NCBI database. The acquired promoter sequences were imported into the JASPAR database to identify binding sites between crucial transcription factors and the promoters of critical lncRNA. Finally, the targeting relationship between them was validated (20).

### Protein-Protein Interaction Analysis of Candidate Target Proteins

The GEO database was utilized to identify microarrays associated with brain neurotoxicity. Specifically, three datasets were chosen: GSE60820, which consists of male Swiss mice cortex samples with five control groups and five experimental groups; GSE212732, which includes male rat cortex samples with four control groups and four experimental groups; and GSE61616, which comprises male rat brain tissue samples with five control groups and five experimental groups. To perform differential analysis, we employed the “limma” package and selected genes with a significance level of *P* < 0.05. For identifying overlapping candidate target proteins associated with brain neurotoxicity, we used the Venn diagram tool from the SangerBox website (http://vip.sangerbox.com/home.html). To assemble their protein-protein interaction networks, we utilized the STRING database (https://string-db.org) with species restriction set to “Homo sapiens.” Finally, we ranked the top 10 nodes in the protein-protein interaction (PPI) network based on their Degree values using the “count” package (21).

### Downstream Target Protein Screening of lncRNA

The amino acid sequences of target genes and the Fasta format sequences of lncRNAs were obtained from the NCBI database (https://www.ncbi.nlm.nih.gov/). Subsequently, the RNA-Protein Interaction Prediction (RPISeq) database (http://pridb.gdcb.iastate.edu/RPISeq/#) was utilized to predict the interactions between lncRNAs and target proteins. Both the random forest (RF) and support vector machine (SVM) signals were employed for screening purposes to identify the crucial target proteins (22).

### Cell Culture and Group Division

SK-N-SH cells (AW-CCH037, Changsha Abiowell Biotechnology) were cultured in DMEM medium (PM150210B, purchased from Wuhan Punoise Life Science) containing 10% fetal bovine serum (FBS) at 37°C and 5% CO_2_ in a constant temperature incubator (23).

### Cell Counting Kit 8 Assay

The vitality of SK-N-SH cells was evaluated using the Cell Counting Kit 8 (CCK-8) assay kit (CA1210) purchased from SoarayBio Technology (Beijing, China). Cells were seeded in a 96-well culture plate at 5 × 10^3^ cells per well in 100 μL of culture medium containing 10% FBS. After allowing the cells to adhere completely, a blank group (containing only culture medium) was set up. Different concentrations of propofol (PPF; PHR1663, purchased from Merck Germany; 0, 25, 50, 100, and 200 μM) were added to the cells at different time points (0, 6, 12, 24, 48 h). Subsequently, 10 μL of CCK-8 solution were added to each well. Results were obtained by measuring the optical density at 490 nm. Each experiment included five replicates, and the entire validation process was repeated three times independently (24).

### Cell Transfection and Grouping

Cell lines with gene knockdown or overexpression were constructed using lentivirus transduction, including sh-NC, sh-RARα, sh-NC + oe-NC, sh-RARα + oe-NC, sh-Shng1 + oe-NC, and sh-RARα + oe-Shng1. Two shRNA sequences were selected for a simultaneous knockdown in the knocking down process, and the group with better effects was chosen for subsequent experiments. The shRNA interference sequences are shown in Table 1. The constructed plasmid was cotransfected with the helper plasmid into 293 T cells to obtain packaged lentivirus after validation, amplification, and purification. Plasmid and lentivirus packaging services are provided by Synbio Technologies (Shanghai, China).

**Table 1.** shRNA Sequence information (human)

Name	shRNA Sequence
shRNA-NC	Sense: 5′-UAAGGCUAUGAAGAGAUACdTdT-3′
	Antisense: 5′-GUAUCUCUUCAUAGCCUUAdTdT-3′
shRNA-RARα-1	Sense: 5′-CAAAGCAAGGCTTGTAGATGC-3′
	Antisense: 5′-GCATCTACAAGCCTTGCTTTG-3′
shRNA-RARα-2	Sense: 5′-TCTTGACAAACAAAGCAAGGC-3′
	Antisense: 5′-GCCTTGCTTTGTTTGTCAAGA-3′
shRNA-Snhg1-1	Sense: 5′-CTAACAGAACCTTTGAATGCC-3′
	Antisense: 5′-GGCATTCAAAGGTTCTGTTAG-3′
shRNA-Snhg1-2	Sense: 5′-GCTAACAGAACCTTTGAATGC-3′
	Antisense: 5′-GCATTCAAAGGTTCTGTTAGC-3′

RARα, retinoic acid receptor-α; Snhg1, small nucleolar RNA host gene 1.

Cells (5 × 10^5^) were plated in a 6-well plate for slow virus-mediated cell transduction and incubated until the cell confluence reached 75%. Then, the medium containing an appropriate amount of packaged slow virus (multiplicity of infection = 10; working titer: ∼5 × 10^6^ TU/mL), and 5 μg/mL polybrene (Merck, TR-1003) were added for transduction. After 4 h of transfection, an equal amount of medium was added to dilute polybrene. After 24 h, it was replaced with fresh medium. After 48 h of transfection, 1 μg/mL puromycin (Thermo Fisher, A1113803) was used to select resistance to obtain a stable cell line. The cells were collected for further experiments after treating SK-N-SH cells with 100 μM propofol for 24 h.

*Cell grouping 1* was as follows: sh-NC + PPF, sh-RARα + PPF, oe-NC + PPF, and oe-RARα + PPF. After transfecting SK-N-SH cells with lentivirus, 100 μM PPF were added for stimulation, and experiments were performed 24 h after stimulation.

*Cell grouping 2* was as follows: PBS (SK-N-SH cells stimulated with PBS for 24 h), PPF (SK-N-SH cells stimulated with 100 μM PPF for 24 h), sh-NC + PPF, and sh-RARα + PPF. After lentivirus transfection of SK-N-SH cells, 100 μM PPF were added for stimulation, and experiments were conducted 24 h after stimulation.

*Cell grouping 3* was as follows: sh-NC + oe-NC + PPF, sh-RARα + oe-NC + PPF, sh-Shng1 + oe-NC + PPF, sh-RARα + oe-Shng1 + PPF, and sh-Shng1 + oe-Bdnf + PPF. After transfection of SK-N-SH cells with lentivirus, 100 μM PPF were added for stimulation, and the experiment was conducted 24 h after stimulation (25). All experiments were independently repeated three times for validation.

### Lactic Acid Dehydrogenase Release Assay

Lactic acid dehydrogenase (LDH) release was measured using the LDH assay kit (BC0680) obtained from Sino Biological (Beijing, China). Different sample groups with varying concentrations (0, 25, 50, 100, and 200 μM) were established and treated in SK-N-SH cells for different time intervals (0, 6, 12, 24, and 48 h). After incubation, the culture plates were centrifuged at 600 *g* for 10 min, and the supernatant (10 µL/well) was transferred to another 96-well plate. Subsequently, 100 µL of LDH reaction mixture were added to each well and incubated at room temperature for 30 min, followed by measuring the absorbance at 490 nm (26). Each experiment was conducted with five parallel holes and repeated independently three times.

### Quantitative RT-PCR

Total RNA from mouse brain tissue and SK-N-SH cells was extracted using TRIzol (15596026, ThermoFisher). The concentration and purity of extracted total RNA were detected using the Nanodrop2000 UV-Vis spectrophotometer (ThermoFisher). Reverse transcription of RNA into cDNA was performed according to the PrimeScript RT reagent Kit (RR047A, Takara, Tokyo, Japan) manual. The synthesized cDNA was then subjected to quantitative RT-PCR (RT-qPCR) analysis using the Fast SYBR Green PCR kit (11736059, ThermoFisher Scientific, Shanghai, China) and corresponding primers. Each hole was set with five replicates, with GAPDH as the internal reference. Calculate relative expression using the 2^−ΔΔCt^ method (26). The experiment was repeated three times. The primer sequences used for RT-qPCR in this study can be found in Table 2.

**Table 2.** RT-qPCR primer sequences

Genes	Primer Sequences (5′-3′)
RARα (human)	Forward: 5′-GAATCGAGCTGAGAGGGCTT-3′
	Reverse: 5′-CCTGTGATGCTGCTCAGGTG-3′
Snhg1(human)	Forword: 5′-GCACGTTGGAACCGAAGAGA-3′
	Reverse: 5′-AATACCTGTATTCACCCTGG-3′
Bdnf (human)	Forword: 5′-CTGGAGCCAGAATCGGAACC-3′
	Reverse: 5′-CTCACCTGGTGGAACTCGG-3′
Gapdh (human)	Forword: 5′-AGGTCGGTGTGAACGGATTTG-3′
	Reverse: 5′-TGTAGACCATGTAGTTGAGGTCA-3′
RARα (mouse)	Forward: 5′-GTTGTTCCTGTCAGCCTGTCTA-3′
	Reverse: 5′-CAAAGAGGATGCCACTCCCAG-3′
Snhg1 (mouse)	Forword: 5′-TTGAGGTGCCACCTTACAAAAG-3′
	Reverse: 5′-TCGAACTCTTCATGTTGTCACAG -3′
Bdnf (mouse)	Forword: 5′-AGCAGAGTCCATTCAGCACC-3′
	Reverse: 5′-GAGCCGAACCTCGGAAAAGA-3′
Gapdh (mouse)	Forword: 5′-CCCACTAACATCAAATGGGG -3′
	Reverse: 5′- ATCCACAGTCTTCTGGGTGG -3′

RARα, retinoic acid receptor-α; Snhg1, small nucleolar RNA host gene 1; Bdnf, brain-derived neurotrophic factor.

### Determination of Reactive Oxygen Species, Superoxide Dismutase, and Malondialdehyde

The cells were seeded in a 96-well culture plate with a density of 5 × 10^3^ cells per well and allowed to fully adhere in 100 μL of culture medium containing 10% FBS. After complete adhesion, a blank group with only a culture medium was set up. Different propofol concentrations (PPF; PHR1663, purchased from Merck, Germany) were used to culture the cells at different time points (0, 6, 12, 24, and 48 h). The levels of reactive oxygen species (ROS), superoxide dismutase (SOD), and malondialdehyde (MDA) were measured using the ROS assay kit (CA1410), SOD assay kit (BC0170), and MDA assay kit (BC0020), respectively. All the assay kits were obtained from Beijing Solabio Company (Solabio Technology, Beijing, China) and used according to the manufacturer’s instructions. All experiments were independently validated by repeating them three times (3).

### Western Blot

The SK-N-SH cells or brain tissues were digested in RIPA buffer (P0013B, Beyotime Biotechnology, Shanghai, China). The protein concentration was quantified using the BCA method with the A53226 assay kit (ThermoFisher Scientific, Rockford, IL). After protein separation, the proteins were transferred to a PVDF membrane (IPVH85R, Millipore, Darmstadt, Germany) using a wet transfer method. The membrane was incubated in 5% BSA at room temperature for 1 h and then with the following primary antibodies: rabbit anti-caspase-3 [Cell Signaling Technology, cat. no. 9662; Research Resource Identifier (RRID): AB_331439; 1:1,000], rabbit anti-cleaved-caspase-3 (Cell Signaling Technology, cat. no. 9654; RRID: AB_10694088; 1:1,000), rabbit anti-RARα (Cell Signaling Technology, cat. no.62294; RRID: AB_2799625; 1:1,000), rabbit anti-Bdnf (Cell Signaling Technology, cat. no.47808; RRID: AB_2894709; 1:1,000), and rabbit anti-Gapdh (Abcam, cat. no. ab9485; RRID: AB_307275; 1:1,000), incubated overnight at 4°C. After being washed, the membrane was incubated with a secondary antibody, goat anti-rabbit IgG horseradish peroxidase conjugate (Abcam, cat. no. ab6721; RRID: AB_955447; 1:5,000), at room temperature for 2 h. The membrane was washed with TBS Tween three times for 5 min each, and chemiluminescence was detected using a chemiluminescence imaging system. Protein quantification analysis was performed using ImageJ 1.48 u software (V1.48, National Institutes of Health), and the protein quantification was determined by the ratio of the grayscale values of each protein to the reference protein β-actin (27). The experiment was repeated three times.

### Flow Cytometry

An annexin V-FITC cell apoptosis detection kit (A211-01) manufactured by the Vazyme Company was utilized to detect apoptosis in SK-N-SH cells. In the experiment, the cells were initially treated with propofol, centrifuged, and resuspended in a binding buffer. Subsequently, annexin V-FITC and propidium iodide (PI) reagents (5 μL each) were introduced into the cells, followed by an incubation period of 15 min. The fluorescently labeled cells were then enumerated using a flow cytometer (FACS Aria) from BD Biosciences, connected to FlowJo 10 software (27).

### Luciferase Reporter Assay

An annexin V-FITC cell apoptosis detection kit (A211-01) manufactured by Vazyme Company was employed to detect cell apoptosis. In the experiment, 5 × 10^6^ SK-N-SH cells were treated with propofol, then centrifuged and resuspended in a binding buffer. Subsequently, annexin V-FITC and PI reagents (5 μL each) were added to the cells and incubated for 15 min. The fluorescently labeled cells were quantified using a flow cytometer (FACS Aria) from BD Biosciences, connected to FlowJo 10 software (28).

### Chromatin Immunoprecipitation

The Simple Chip Enzymatic Chromatin IP Kit (9003, Cell Signaling Technology) was used for chromatin immunoprecipitation (ChIP) analysis according to the manufacturer’s instructions. In brief, formaldehyde was used to cross link 5 × 10^6^ SK-N-SH cells, followed by sonication to generate 200- to 1,000-bp fragments. Chromatin immunoprecipitation was performed using 3 μg of IgG antibody (Cell Signaling Technology, cat. no. 62294; RRID: AB_2799625; 1:50) against RARα in neuronal chromatin. The immunoprecipitated DNA was then subjected to RT-qPCR analysis using specific primers (28). The primer sequence for the Snhg1 promoter can be found in Table 3.

**Table 3.** RT-qPCR Primer sequences

Genes	Primer Sequences(5′-3′)
Snhg1(promoter)	Forward:5′-GGCAGCTCAGAACAGGGTTA-3′
	Reverse:5′-AAGGAGGGCGTAGACAAAGC-3′

RARα, retinoic acid receptor-α; Snhg1, small nucleolar RNA host gene 1.

### Constructing a Mouse Neurotoxicity Model

C57BL/6 mice (males, 6 wk old, strain code 219, purchased from Vetonlihua Laboratory Animal Technology. Beijing, China) were prefed for 7 days. The mice were randomly divided into six groups, with eight in each group. The groups were named Saline, PPF, sh-NC + oe-NC + PPF, sh-RARα + oe-NC + PPF, sh-Shng1 + oe-NC + PPF, and sh-RARα + oe-Shng1 + PPF. Please refer to Table 4 for the transfection sequence. The Saline group was intravenously injected with 0.9% saline solution via the tail vein daily. The PPF group, sh-NC + oe-NC + PPF group, sh-RARα + oe-NC + PPF group, sh-Shng1 + oe-NC + PPF group, and sh-RARα + oe-Shng1 + PPF group were intravenously injected with 5 mg/kg propofol via the tail vein for 7 consecutive days. The sh-NC + oe-NC + PPF group, sh-RARα + oe-NC + PPF group, sh-Shng1 + oe-NC + PPF group, and sh-RARα + oe-Shng1 + PPF group were used to overexpress or suppress RARα and Shng1 using lentiviral-mediated sh-RARα, sh-Shng1, and oe-Shng1 vectors, respectively.

**Table 4.** shRNA sequence information (rat)

Name	shRNA Sequence
shRNA-NC	Sense: 5′-UAAGGCUAUGAAGAGAUACdTdT-3′
	Antisense: 5′-GUAUCUCUUCAUAGCCUUAdTdT-3′
shRNA-RARα-1	Sense: 5′-TGTTGTTCGTAGTGTACTTGC-3′
	Antisense: 5′-GCAAGTACACTACGAACAACA-3′
shRNA-RARα-2	Sense: 5′-ATTTCCTGGATAAGTGGTGGC-3′
	Antisense: 5′-GCCACCACTTATCCAGGAAAT-3′
shRNA-Snhg1-1	Sense: 5′-CTAACAGAACCTTTGAATGCC-3′
	Antisense: 5′-GGCATTCAAAGGTTCTGTTAG-3′
shRNA-Snhg1-2	Sense: 5′-GCTAACAGAACCTTTGAATGC-3′
	Antisense: 5′-GCATTCAAAGGTTCTGTTAGC-3′

RARα, retinoic acid receptor-α; Snhg1, small nucleolar RNA host gene 1.

The vectors were delivered using stereotactic injection via predrilled cranial holes into the ventricle, with a dose of 1 × 10^8^ viral particles reconstituted in 5 μL of PBS, injected into the ventricle on the first day post-PPF injection (29). The procedure was performed as described in a previously published article (30). In summary, mice were anesthetized in an induction chamber using 4% isoflurane. Following anesthesia, the heads were shaved. The mouse’s tongue was gently moved using forceps and placed in a stereotaxic instrument. An ear bar was inserted and secured. The mouse was positioned on a heating pad with the nose cone inserted. Then, 4% isoflurane was administered through the nose cone. A toe pinch confirmed anesthesia.

The eyes were lubricated, and the shaved head was prepared using alternating povidone-iodine and lidocaine swabs for injection site preparation. A surgical blade made a small incision in the scalp’s center. The anterior fontanelle was visualized using a dissecting microscope, and the drill tip was centered over the fontanelle and zeroed (or recorded) digital coordinates. The head was leveled along the rostrocaudal *y*-axis, and the drill tip was placed on lambda. The *z*-coordinate was approximately equal between bregma and lambda. The drill tip was positioned at the desired coordinates, and for the dentate gyrus, the coordinates from the anterior fontanelle were *y* = −1.9 and *x* = ±1.1. The isoflurane concentration was reduced to 2% before drilling, and the cranial bone was carefully perforated.

After all holes were drilled, the filled syringe was secured to the instrument. The syringe was aligned with the holes, and the *z*-coordinate at the skull was zeroed. The syringe was slowly lowered to the deepest *z*-depth, which was −2.5, −2.4, and −2.3 for the dentate gyrus. The injection was started at a rate of 0.25 μL/min using the stereotactic injector. After injection, the mouse was removed, and the scalp was sutured with 6-0 silk thread. Lidocaine and antimicrobial ointment were applied. The intraperitoneal injection of 0.8–1.0 mL of saline and ketoprofen (3–5 mg/kg) controlled pain. The mouse was then placed in a recovery area.

All animal experiments strictly adhered to the *Guidelines on the Care and Use of Laboratory Animals* set by the National Institute of Health and have been approved by our institutional Animal Ethics Committee.

### Morris Water Maze Experiment

The Morris water maze comprises a circular water pool with a diameter of 150 cm and a depth of 20 cm. After the addition of carbon ink, the water in the pool becomes opaque black. The water surface is 0.5 cm higher than the platform surface, dyed black to make it invisible to the mice. The temperature of the water is 22–25°C. During the acquisition phase, mice are trained for six consecutive days with four sessions per day to learn how to swim to the platform in the water. Each mouse’s starting point is randomly assigned, and the mice are allowed 60 s to find the platform hidden in the water. If the mouse fails to locate the platform within 60 s, researchers will manually guide it to the platform and allow it to stay there for 20 s. The time it takes for a mouse to reach the platform could reflect spatial learning ability and is called escape latency. On the seventh day, the researchers conduct a probing experiment, remove the platform, and allow all mice to swim for 60 s, recording the number of times the mice cross the platform’s location (31, 32).

### Terminal Deoxynucleotidyl Transferase dUTP Nick End Labeling Staining

Cell apoptosis in brain tissue paraffin sections was assessed using the terminal deoxynucleotidyl transferase dUTP nick end labeling (TUNEL) staining kit. The 7-μm paraffin sections of the cerebral cortex tissue were subjected to deparaffinization and rehydration. Subsequently, the tissue sections were incubated in Tris buffer solution (pH 8) containing 15.3 mg/mL proteinase K for 20 min, followed by washing in 50 mM TBS (pH 7.6). The sections were then incubated with the enzyme solution (C1086, Bioss, Shanghai, China) labeled with green fluorescence and subsequently incubated with the culture medium containing 4′,6′-diamidino-2-phenylindole (DAPI) for 10 min. TUNEL-positive cells were identified using a fluorescence microscope (Leica Microsystems, Wetzlar, Germany), in which TUNEL-positive cells appeared green and DAPI staining appeared blue, indicating nuclear morphology. Furthermore, the staining positivity rate was calculated (33).

### Immunofluorescent Staining of Immune Cells

After dewaxing treatment of the brain cortex tissue slices, the slices were immersed in 3% H_2_O_2_ for 10 min to inhibit endogenous peroxidase activity. Subsequently, the slides were immersed in antigen retrieval solution with a pH of 6.0 and heated in a microwave for 15 min to expose the antigen. A protein-blocking solution was added to the tissue to prevent nonspecific binding. BDNF antibody (Abcam, cat. no. ab108319, RRID: AB_10862052) was applied to the slices at a dilution of 1:500. Incubation was carried out overnight at 4°C, followed by a 2-h incubation at 37°C with a mixture of FITC and TRITC-conjugated secondary antibodies. Finally, the stained slices were examined using a fluorescence microscope (Zeiss Scope A1) (34).

### Statistical Analysis

All data were processed using GraphPad Prism 8.0.2 statistical software. The measurement data are represented as means ± SD. For the comparison between the two groups, the unpaired *t* test is employed. For comparisons between multiple groups, one-way ANOVA was used. *P* < 0.05 was a statistically significant difference.

## RESULTS

### Propofol-Induced Toxicity in SK-N-SH Cells

**Figure 1.**
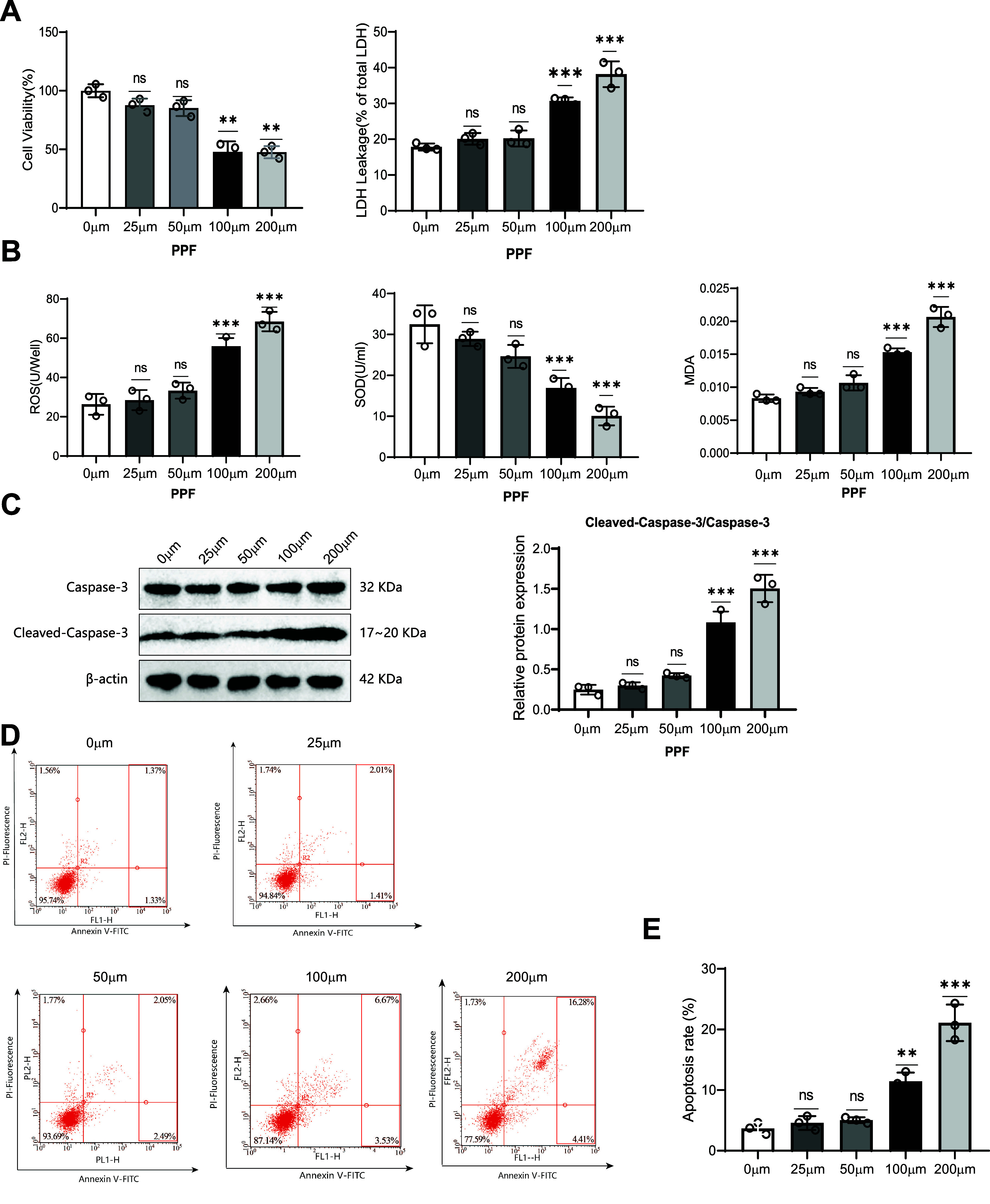
Effects of different propofol concentrations on the viability, oxidative stress, and apoptosis of SK-N-SH cells. The viability, oxidative stress, and apoptosis of SK-N-SH cells were measured after treatment with different concentrations (0, 25, 50, 100, and 200 μM) of propofol (PPF) for 24 hours. *A*: viability and lactic acid dehydrogenase (LDH) release. *B*: reactive oxygen species (ROS) level, SOD activity, and malondialdehyde (MDA) content. *C*: expression of cleaved caspase-3 protein. *D* and *E*: apoptosis rate of cells and representative flow cytometry plots for each group. **P < 0.01, significant difference compared to the 0-μM group; ***P < 0.001, significant difference compared to the 0-μM group; ns, no significant difference; all experiments were repeated 3 times.

**Figure 2.**
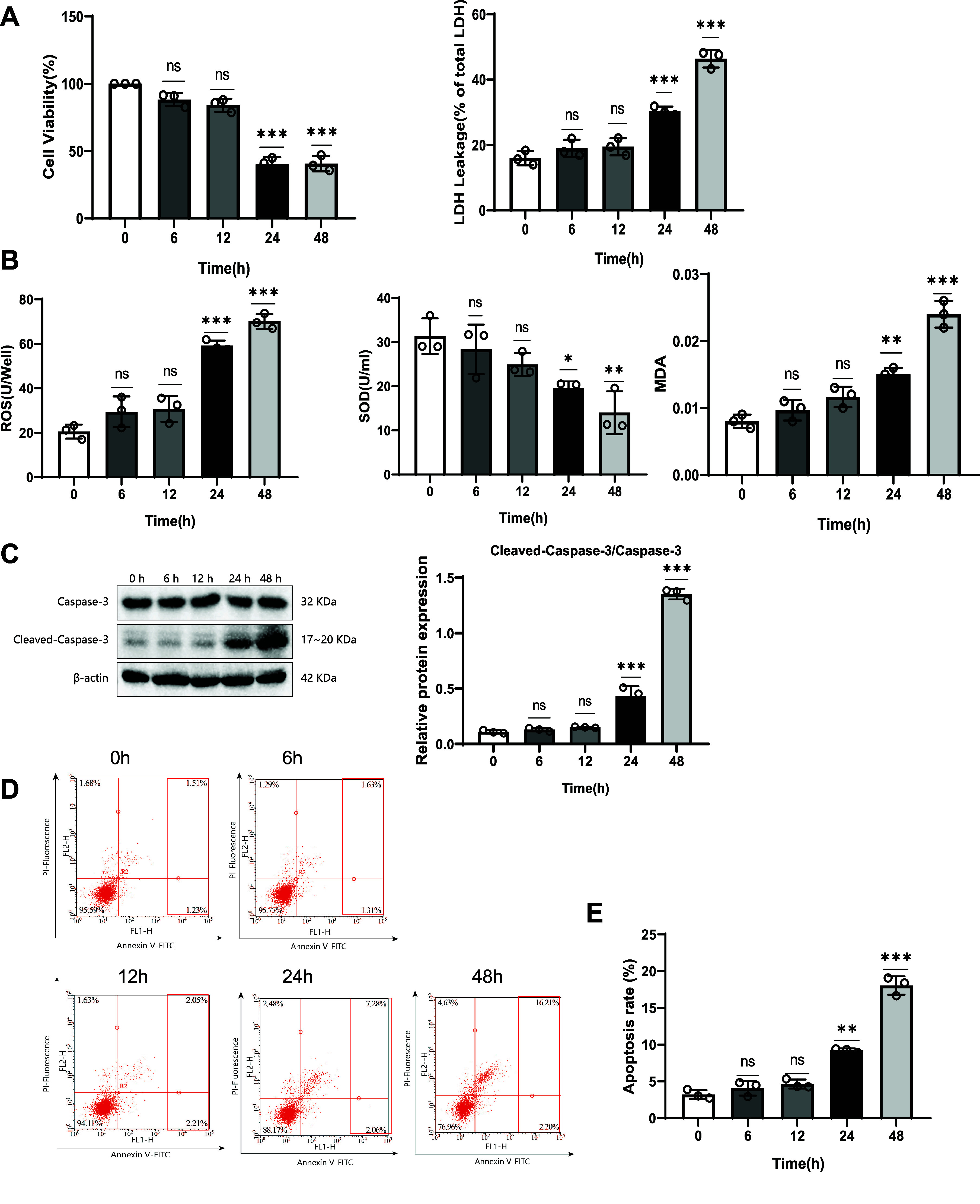
Effects of different treatment durations on SK-N-SH cells’ viability, oxidative stress, and apoptosis. SK-N-SH cells were treated with 100 μM propofol (PPF) for different durations (0, 6, 12, 24, 48 h), and then the viability, oxidative stress, and apoptosis were measured. *A*: viability (Cell Counting Kit 8 assay) and lactic acid dehydrogenase (LDH) release. *B*: reactive oxygen species (ROS) level, SOD activity, and malondialdehyde (MDA) content. *C*: expression of cleaved caspase-3 protein. *D* and *E*: apoptosis rate of cells and representative flow cytometry plots for each group. PI, propidium iodide. *P < 0.05, significant difference compared to the 0-h group; **P < 0.01, significant difference compared to the 0-h group; ***P < 0.001, significant difference compared to the 0-h group; ns, no significant difference; all experiments were repeated 3 times.

The intravenous anesthetic propofol (PPF) has been shown to induce neurotoxicity, leading to neuronal apoptosis and oxidative stress in the brain (35, 36). To evaluate the neurotoxicity of propofol, we assessed the viability, oxidative stress, and apoptosis in SK-N-SH cells. Initially, the cells were treated with different propofol concentrations (0, 25, 50, 100, and 200 μM) for 24 h. The results indicated that cell viability remained unchanged at propofol concentrations of 25 and 50 μM. However, at a concentration of 100 μM, SK-N-SH cell viability significantly decreased, accompanied by a marked increase in LDH release. The levels of reactive oxygen species (ROS) significantly increased, while superoxide dismutase (SOD) activity significantly decreased, and malondialdehyde (MDA) content significantly increased. Moreover, the expression of cleaved caspase-3 increased significantly, along with a notable increase in cellular apoptosis rate (Fig. 1). Subsequently, we selected a propofol concentration of 100 μM to treat SK-N-SH cells for different durations (0, 12, 24, and 48 h). The results showed that cell viability remained unchanged after 12 h of propofol treatment. However, after 24 h, the viability of SK-N-SH cells significantly decreased, accompanied by a marked increase in LDH release. Additionally, ROS levels significantly increased, while SOD activity significantly decreased, and MDA content significantly increased. The expression of cleaved caspase-3 also increased significantly, with a notable increase in cellular apoptosis rate (Fig. 2). These results collectively indicate that propofol impairs SK-N-SH cells and induces neurotoxicity. Therefore, we chose to treat SK-N-SH cells with a concentration of 100 μM for 24 h to obtain optimal effects.

### Selection of Critical Genes and Transcription Factors Related to Neurotoxicity Induced by Propofol in the Brain

**Figure 3.**
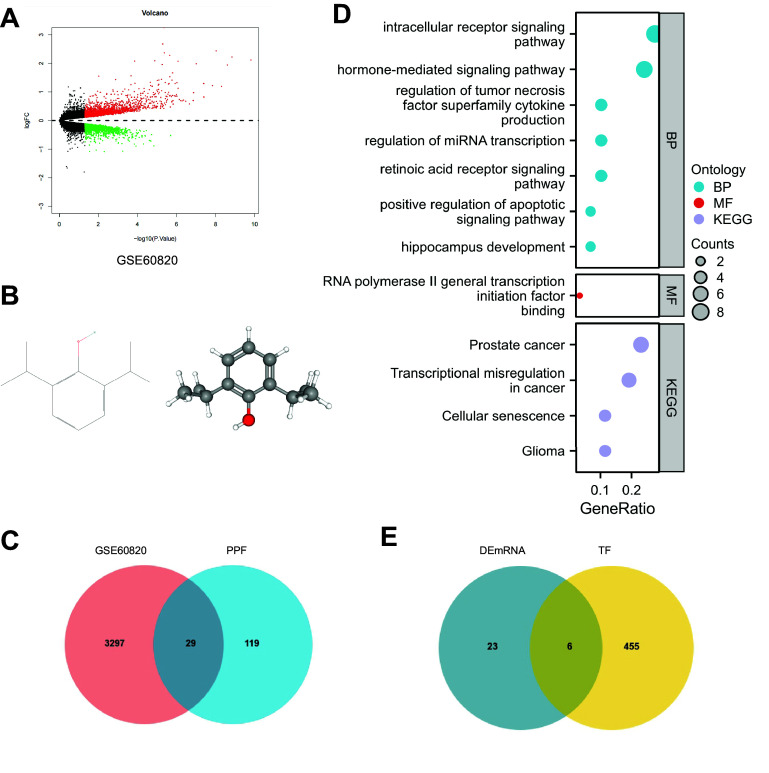
Screening of essential target genes and target transcription factors for propofol (PPF)-induced neurotoxicity in the brain. Bioinformatics analysis was conducted to screen essential target genes for propofol-induced neurotoxicity in the brain. *A*: volcano plot showing differentially expressed genes in GSE60820, with red indicating upregulated genes, and green indicating downregulated genes, number of samples in control group and experimental group: *n* = 5. *B*: 2-dimensional and 3-dimensional structures of propofol. *C*: Venn diagram showing the overlap between differentially expressed genes in GSE60820 and target genes of propofol. *D*: Gene Ontology (GO) and Kyoto Encyclopedia of Genes and Genomes (KEGG) enrichment analysis of target genes for propofol-induced neurotoxicity in the brain, circle size represents the number of genes enriched in each pathway. BP, biological processes; MF, molecular functions. *E*: Venn diagram showing the overlap between target genes for propofol-induced neurotoxicity in the brain and transcription factors (TF).

**Figure 4.**
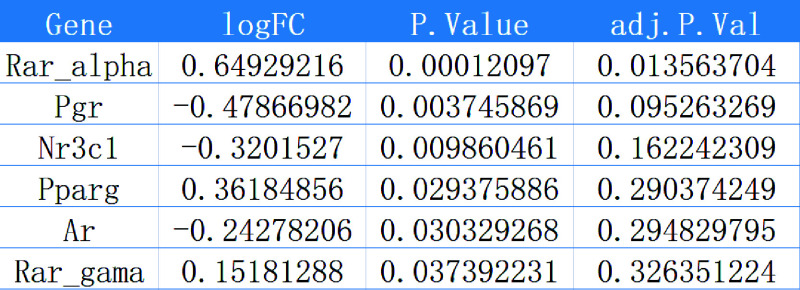
Differential expression matrix of candidate transcription factors in GSE60820. The logFC value represents upregulation if positive and downregulation if negative in the expression matrix, P.Value represents the *P* value, and adj.P.Val represents the adjusted *P* value.

To explore the target of propofol-induced neurotoxicity in the brain, we first screened the neurotoxicity-related chips and obtained 3,326 differentially expressed genes (DEGs), including 1786 upregulated DEGs and 1,540 downregulated DEGs from GSE60820 (Fig. 3*A*). Furthermore, propofol’s 2-D and 3-D structures (Fig. 3*B*) were obtained from the PubChem database, and the propofol target genes were analyzed and screened using the PharmMapper server. A total of 149 relevant genes were selected. The Venn analysis of these target genes with GEO data identified 29 propofol target genes associated with brain neurotoxicity regulation (Fig. 3*C*). The 29 genes were subjected to GO-KEGG pathway enrichment analysis, and the results revealed that propofol-induced neurotoxicity in the brain was associated with the retinoic acid receptor (RAR) signaling pathway, miRNA transcription, regulation of tumor necrosis factor superfamily cytokine production, hippocampal development, positive regulation of apoptosis signaling pathway, binding of RNA polymerase II general transcription initiation factors, and cellular senescence, which are related to biological processes, molecular functions, and signaling pathways (Fig. 3*D*). We used the hTFtarget database to obtain a transcription factor dataset and performed a Venn analysis between the dataset and the 29 potential target genes. As a result, we identified six candidate transcription factors (RARα, Pgr, Nr3c1, Pparg, Ar, and RARγ) that are potentially involved in propofol-induced neurotoxicity in the brain (Fig. 3*E*). RARα is significantly expressed in the DEGs (Fig. 4) among these six transcription factors. In the GO-KEGG pathway enrichment analysis, we also confirmed that propofol-induced neurotoxicity in the brain enriched the RAR signaling pathway (GO:0048384). In addition, studies have shown that RARα could regulate the transcription activity of proteins by binding to the promoter (37). Therefore, we preliminarily conclude that RARα is a crucial transcription factor for propofol-induced neurotoxicity in the brain.

### Propofol Upregulates RARα and Induces Toxicity in SK-N-SH Cells

**Figure 5.**
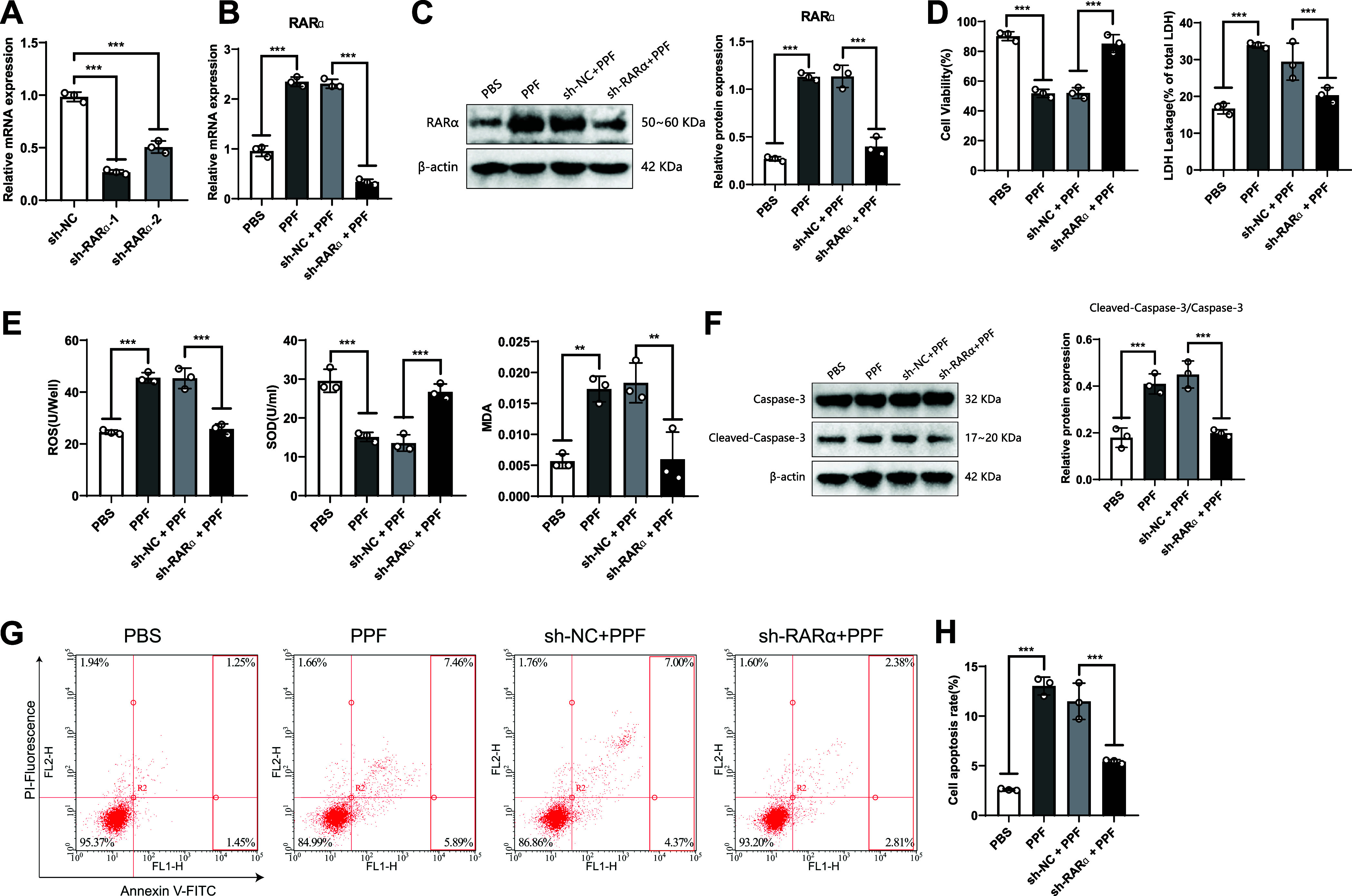
Propofol regulates retinoic acid receptor-α (RARα) to induce toxicity in SK-N-SH cells. Knockdown of RARα was performed to validate the regulation of RARα by propofol (PFF) in inducing toxicity in SK-N-SH cells. *A*: quantitative RT-PCR (RT-qPCR) detection of RARα expression in SK-N-SH cells before and after RARα knockdown. *B*: RT-qPCR detection of RARα gene expression. *C*: Western blot analysis of RARα protein expression. *D*: cell viability and lactic acid dehydrogenase (LDH) release rate. *E*: reactive oxygen species (ROS) levels, SOD activity, and malondialdehyde (MDA) content. *F*: expression of cleaved-caspase-3 protein. *G* and *H*: cell apoptosis rate and representative flow cytometry plots of each group. PI, propidium iodide. Multiple group comparisons were analyzed using ANOVA. **P < 0.01; ***P < 0.001; all experiments were repeated 3 times.

Through bioinformatics analysis, we found that propofol upregulates RARα, leading to neurotoxicity in the brain. To confirm this finding, we used lentivirus to silence RARα (Fig. 5*A*), and sh-RARα-1 was selected for subsequent experiments due to its better-interfering effect. The results indicate that compared with the PBS group, the RARα gene and protein expression in the PPF group were significantly upregulated (Fig. 5, *B* and *C*). However, compared with the sh-NC + PPF group, the RARα gene and protein expression was significantly downregulated in the sh-RARα + PPF group. This result indicates that propofol could promote the expression of RARα, and the sh-RARα + PPF group successfully knocked out the RARα gene.

In addition, we also observed whether the silencing of the RARα gene affects the toxicity of propofol-induced SK-N-SH cells. We measured cellular activity, oxidative stress, and apoptosis-related markers. The results show no significant difference between the PPF and sh-NC + PPF groups. However, compared to the sh-NC + PPF group, the sh-RARα + PPF group showed significantly increased cell viability and decreased LDH release. In addition, the ROS level decreased, SOD activity increased, and MDA content also decreased significantly. It indicates that propofol induces toxicity in SK-N-SH cells by upregulating RARα. Regarding cell apoptosis, the expression of cleaved caspase-3 and the cell apoptosis rate was significantly decreased in the sh-RARα + PPF group (Fig. 5, *D* and *H*).

### RARα Activates the Transcription of Snhg1 Expression

**Figure 6.**
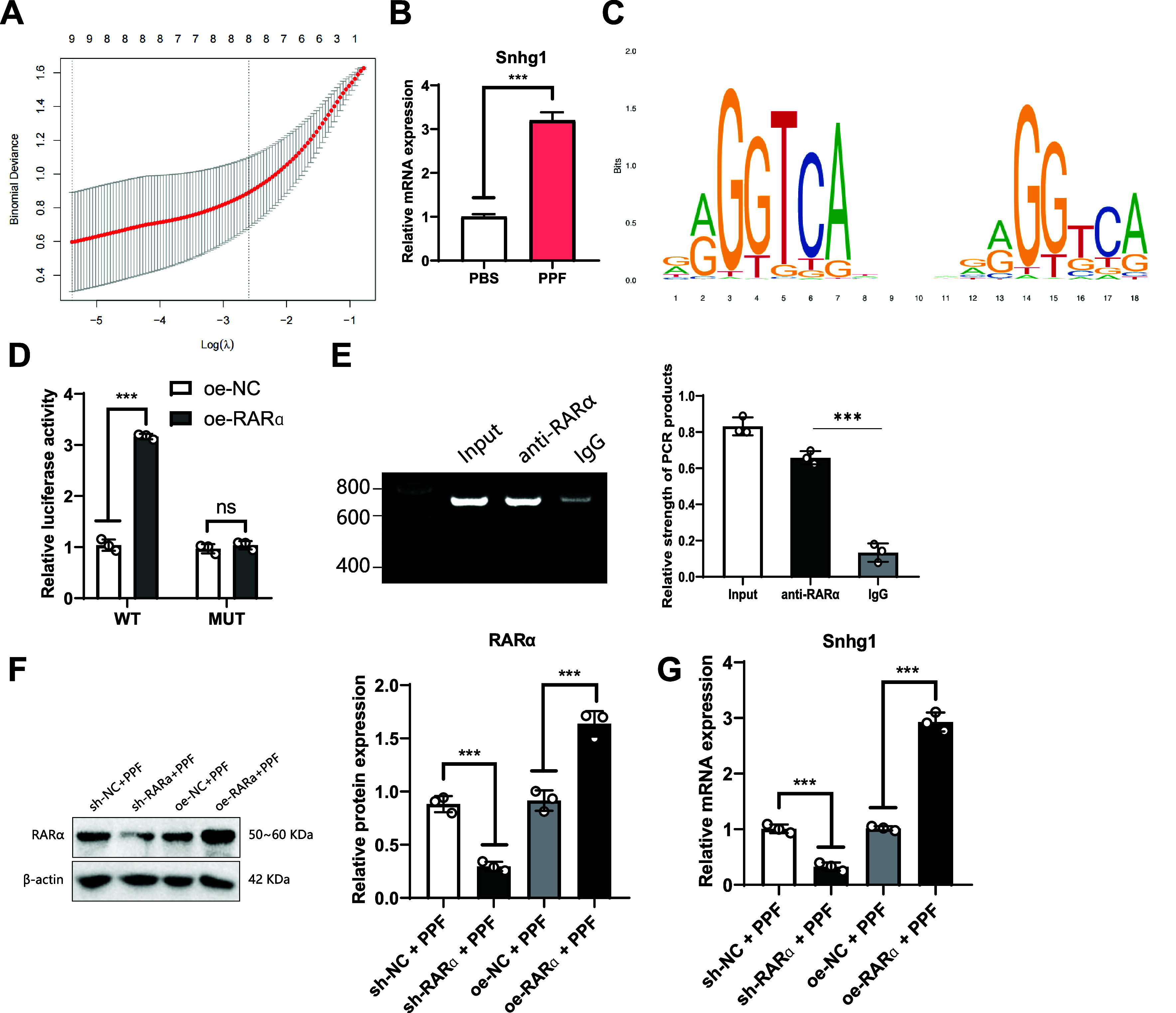
Transcriptional regulation of small nucleolar RNA host gene 1 (Snhg) by retinoic acid receptor-α (RARα). RARα binds to the promoter region of Snhg1 to regulate its expression. *A*: Lasso machine learning for selecting disease-associated long noncoding RNA features. *B*: quantitative RT-PCR (RT-qPCR) detection of Snhg1 gene expression in propofol (PFF)-induced SK-N-SH cells. *C*: JASPAR prediction of binding sites between RARα and Snhg1. *D*: dual-luciferase reporter assay to measure relative luciferase activity of RARα in the promoter region of Snhg1. WT, wild type; MUT, mutant. *E*: chromatin immunoprecipitation (ChIP) analysis of RARα enrichment in the promoter region of Snhg1. *F*: Western blot analysis of RARα protein expression. *G*: RT-qPCR detection of Snhg1 gene expression; Multiple group comparisons were analyzed using ANOVA. ***P < 0.001; ns, no significant difference; all experiments were repeated 3 times.

**Figure 7.**
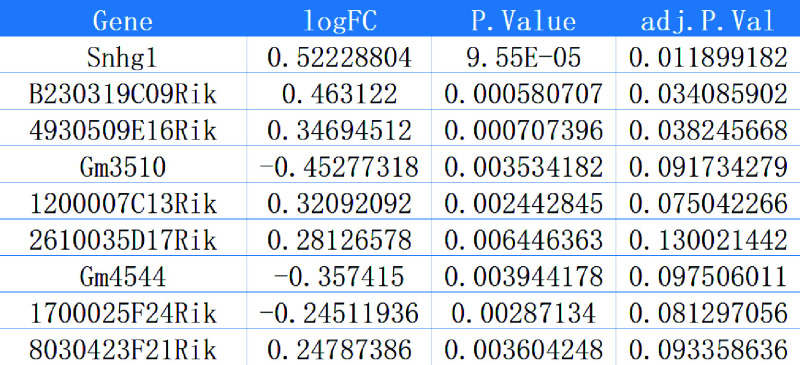
Differential expression matrix of the characteristic long noncoding RNAs in GSE60820. The logFC value represents upregulation if positive and downregulation if negative in the expression matrix, P.Value represents the *P* value, and adj.P.Val represents the adjusted *P* value.

In previous studies, we found that the regulatory effect of lncRNA affects cell apoptosis induced by propofol (38). Meanwhile, research has shown that transcription factors could regulate lncRNA expression by binding to the lncRNA promoter (37). We classified the differentially expressed genes (DEGs) in GSE60820 into categories and found 124 differential lncRNAs. We performed multivariable Cox analysis of differential lncRNAs in GSE60820 using LASSO regression and extracted 9 featured lncRNAs (Fig. 6*A*), including Snhg1, B230319C09Rik, 4930509E16Rik, Gm3510, 1200007C13Rik, 2610035D17Rik, Gm4544, 1700025F24Rik, and 8030423F21Rik. Snhg1 is the most significant feature gene (Fig. 7). Studies show that high expression of Snhg1 promotes disease progression and cell apoptosis (39). Therefore, we selected Snhg1 as a candidate lncRNA. In addition, we found that the expression level of the Snhg1 gene showed a significant increase after 24 h of PPF treatment in SK-N-SH cells (Fig. 6*B*).

**Figure 8.**
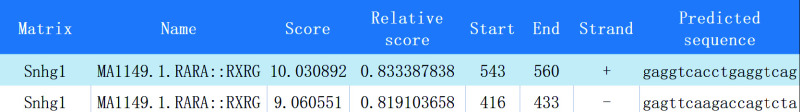
JASPAR predicted binding scores of retinoic acid receptor-α (RARα) and small nucleolar RNA host gene 1 (Snhg1).

Some studies have shown that transcription factors could bind to specific promoter regions to regulate gene expression. Therefore, we investigated whether RARα could regulate the expression level of Snhg1 by binding to the promoter region of Snhg1. We found the promoter sequence of Snhg1 in the NCBI database and used the JASPAR database to predict the binding sites of RARα with the key Snhg1 promoter (Fig. 6*C*; Fig. 8).

We performed a luciferase reporter gene assay to determine whether RARα binds to the Snhg1 promoter. Specifically, we transfected HEK293T cells with luciferase vectors containing either the promoter region sequence of Snhg1 or the sequence of RARα. The results showed that in the oe-RARα group, the luciferase activity was significantly increased compared to the oe-NC group, while there was no significant difference in the MUT group (Fig. 6*D*).

We also used a ChIP assay to validate the binding of RARα and Snhg1 promoter regions. The results indicated that in the RARα immunoprecipitation, we observed PCR products, confirming the binding of RARα to the Snhg1 promoter (Fig. 6*E*).

We knocked down or overexpressed the RARα gene in SK-N-SH to validate our hypothesis further. Western blot results showed successful knockdown or overexpression of RARα (Fig. 6*F*). The results of RT-qPCR showed that the expression of Snhg1 was significantly decreased after knocking down RARα, while the expression of Snhg1 was significantly increased after overexpressing RARα (Fig. 6*G*). It indicates that RARα activates the transcription of Snhg1 expression. In addition, the expression levels of RARα and Snhg1 were upregulated in GSE60820, further confirming the crucial signaling cascade of RARα transcription promoting lncRNA Snhg1 expression in propofol-induced neurotoxicity in the brain.

### Propofol-Induced Central Nervous System Neurotoxicity Core Protein Screening

**Figure 9.**
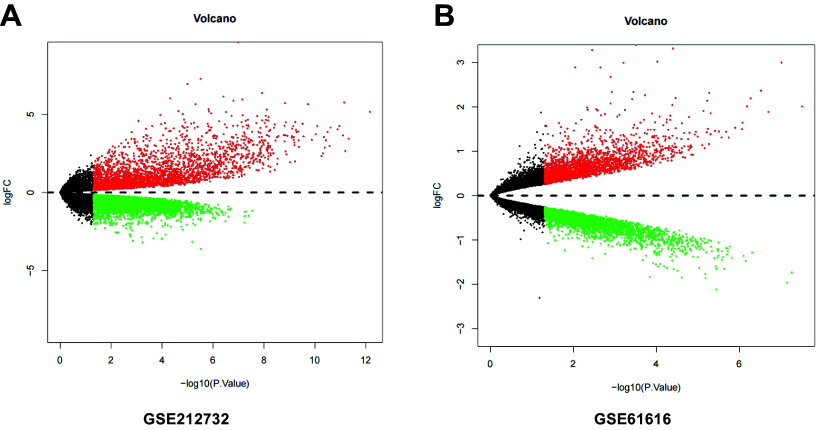
Bioinformatics-based screening of core proteins involved in propofol (PFF)-induced neurotoxicity in the brain. *A*: volcano plot of differentially expressed genes in GSE212732. *B*: volcano plot of differentially expressed genes in GSE616162. Red indicates upregulated genes, green indicates downregulated genes, and black indicates genes with no significant difference (*P* > 0.05).

**Figure 10.**
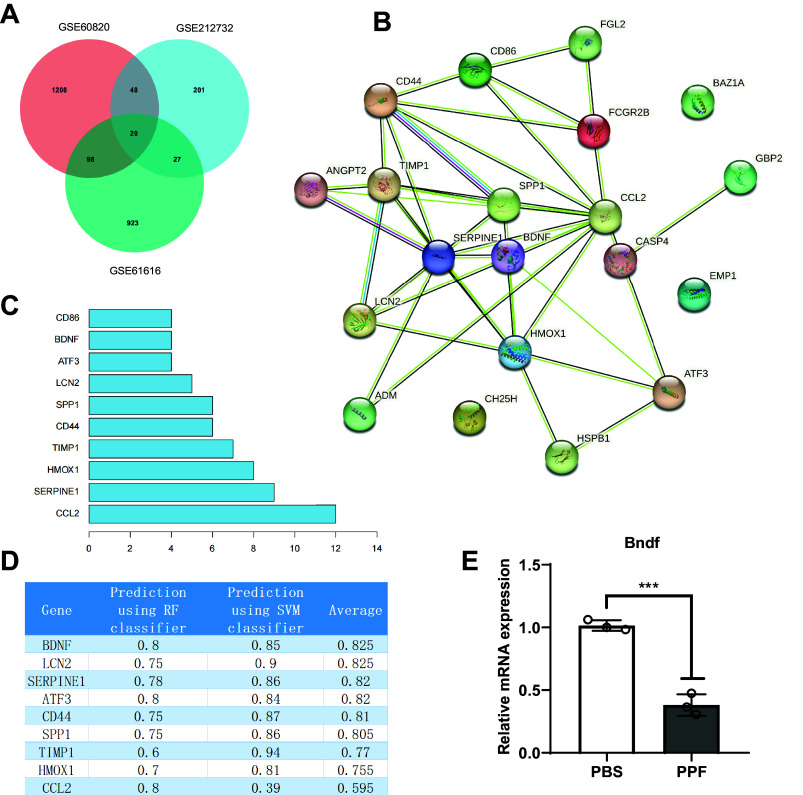
Bioinformatics screening of core proteins induced by propofol in neurotoxicity in the brain. Bioinformatics methods were employed to screen core proteins. *A*: Venn analysis of GSE60820, GSE212732, and GSE61616. *B*: interaction network of proteins encoded by the 20 target genes for propofol-induced neurotoxicity in the brain, with each node representing a protein and the edges representing protein-protein interactions. *C*: display of the top 10 target proteins in the degree values of the 20 target proteins encoded by propofol-induced neurotoxicity in the brain, with the *y*-axis representing proteins and the *x*-axis representing degree values. *D*: scores from RNA-Protein Interaction Prediction (RPISeq) database for RNA-protein interaction predictions of Snhg1 and the target genes encoded proteins. RF, random forest; SVM, support vector machine. *E*: quantitative RT-PCR detection of brain-derived neurotrophic factor (Bdnf) gene expression in propofol (PFF)-induced SK-N-SH cells. The data were analyzed using an independent samples *t* test for statistical analysis. *P* < 0.001, compared to the 2 groups; all experiments were repeated 3 times.

In the previous context, we have identified that propofol induces neuronal toxicity in the brain through the transcriptional effect of RARα promoting Snhg1 expression. To further clarify the downstream regulatory mechanism of RARα-Snhg1, we screened the core protein expression related to propofol-induced neurotoxicity in the brain. We confirmed that propofol regulates the expression of these proteins through the RARα-Snhg1 pathway, thereby inducing neurotoxicity in the brain. We selected two sets of related chips, GSE61616 and GSE212732, from the GEO public database and performed differential analysis, resulting in 1068 and 296 differential mRNAs (Fig. 9). By performing Venn analysis on the differentially expressed mRNA identified by GEO60820 screening, we obtained 20 differentially expressed mRNAs (Fig. 10*A*). Furthermore, we conducted PPI network analysis on these 20 differentially expressed mRNAs using the STRING database, identifying 10 core genes related to brain neurotoxicity. The 10 core genes are Ccl2, Serpine1, Hmox1, Timp1, CD44, Spp1, Lcn, Atf3, Bdnf, and CD86 (Fig. 10*B*). Figure 10*C* shows the Degree values of these 10 genes. Based on this, we used the RPISeq database to predict the binding of genes to Snhg1, using two methods, RF classifier and SVM classifier, to predict the binding probability of Snhg1 and 10 core genes. It was found that Snhg1 has the potential to interact with the proteins encoded by these genes, and the protein with the highest and most stable prediction probability is Bdnf (Fig. 10*D*). It is consistent with the literature reports where Bdnf plays an important role in brain neurotoxicity (40). In addition, we also observed a significant decrease in the expression level of the Snhg1 gene in SK-N-SH cells after PPF treatment (Fig. 10*E*). The literature reports the negative regulation of protein expression by Snhg1 (41). Based on this, we preliminarily infer that RARα binds to the promoter region of lncRNA Snhg1 to initiate brain neurotoxicity by suppressing Bdnf expression, forming a crucial regulatory network.

### Propofol Induces Toxicity in SK-N-SH Cells via RARα-Snhg1-Bdnf

**Figure 11.**
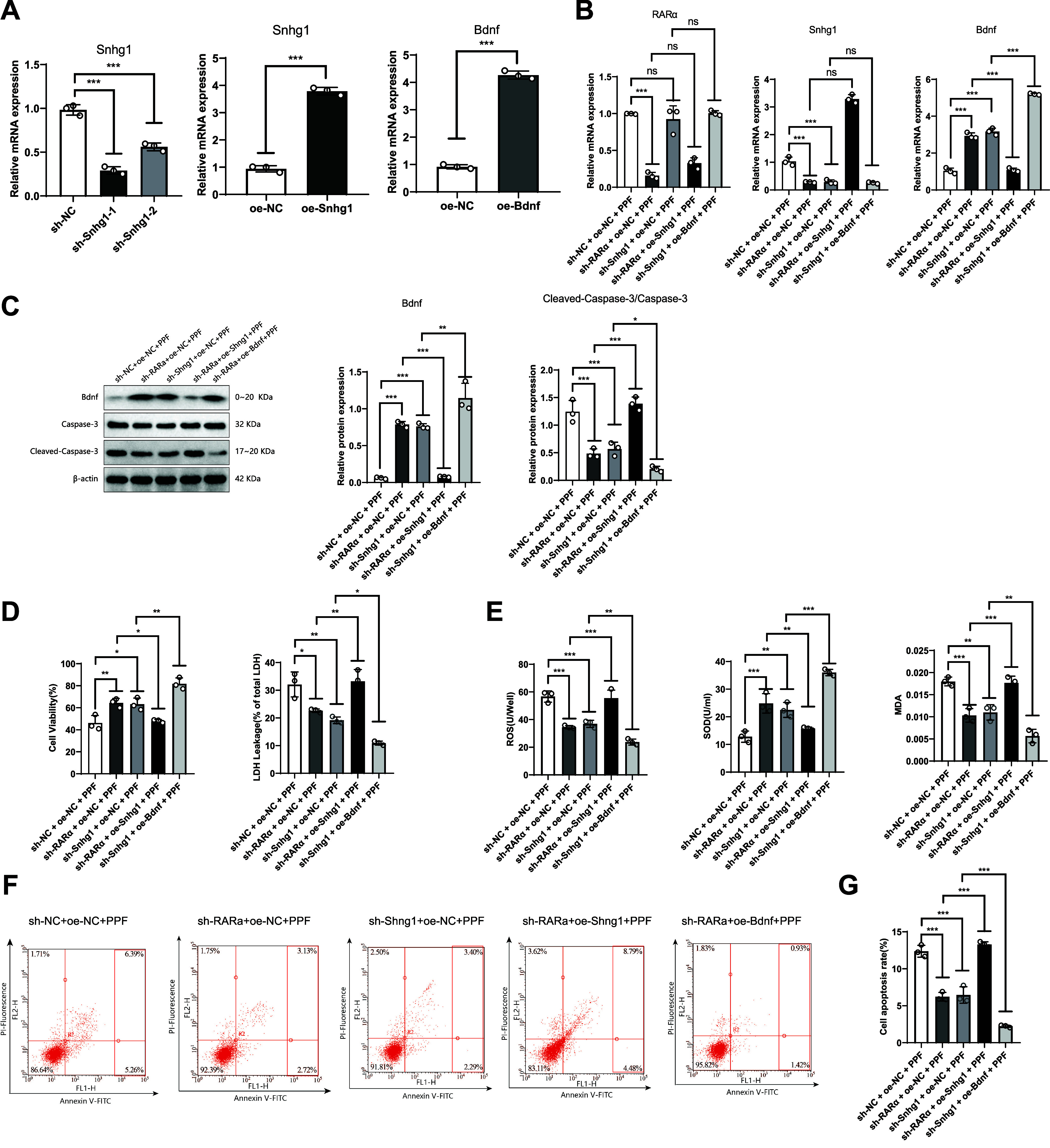
Propofol promotes SK-N-SH toxicity through the retinoic acid receptor-α, small nucleolar RNA host gene 1, and brain-derived neurotrophic factor (RARα-Snhg1-Bdnf) pathway. Knockdown or overexpression of RARα and Snhg1 affect propofol-induced cell viability, oxidative stress, and apoptosis in SK-N-SH cells. *A*: quantitative RT-PCR detection of Snhg1 and Bdnf expression in SK-N-SH cells before and after knockdown or overexpression of Snhg1 or Bdnf. *B*: gene expression of RARα, Snhg1, and Bdnf. *C*: protein expression of Bdnf and cleaved-caspase-3. *D*: cell viability and lactic acid dehydrogenase (LDH) release rate. *E*: reactive oxygen species (ROS) levels, SOD activity, and malondialdehyde (MDA) content. *F* and *G*: cell apoptosis rate and representative flow cytometry plots of each group. PI, propidium iodide. *P < 0.05, in comparison between 2 groups; **P < 0.01; ***P < 0.001; ns, no significant difference; all experiments were repeated 5 times.

To investigate whether propofol induces neuronal toxicity through the RARα-Snhg1-Bdnf pathway, we used lentiviruses to separately knock down RARα and Snhg1 and overexpress Snhg1 and Bdnf (Fig. 11*A*). We detected gene expression of RARα, Shng1, and Bdnf using RT-qPCR. The results showed that compared to the sh-NC + oe-NC + PPF group, the RARα and Shng1 expression was significantly downregulated in the sh-RARa + oe-NC + PPF group, while the Bdnf expression was significantly upregulated. In the sh-Shng1 + oe-NC + PPF group, the Shng1 expression was significantly downregulated, while the Bdnf expression was significantly upregulated. Compared to the sh-RARa + oe-NC + PPF group, the sh-RARa + oe-Shng1 + PPF group showed a significant upregulation of Shng1 expression, while Bdnf expression was downregulated. Compared to the sh-Shng1 + oe-NC + PPF group, the sh-Shng1 + oe-Bndf + PPF group showed a significant upregulation in Bdnf expression (Fig. 11*B*).

We also used Western blot to detect Bdnf, caspase-3, and cleaved-caspase-3 protein expression. The results showed that compared to the sh-NC + oe-NC + PPF group, the sh-RARa + oe-NC + PPF group, and the sh-Shng1 + oe-NC + PPF group significantly promoted the expression of Bdnf protein and reduced the ratio of cleaved-caspase-3 to caspase-3. Compared to the sh-RARa + oe-NC + PPF group, the sh-RARa + oe-Shng1 + PPF group significantly inhibits Bdnf protein expression and increases the cleaved-caspase-3:caspase-3 ratio. Compared to the sh-Shng1 + oe-NC + PPF group, the sh-Shng1 + oe-Bndf + PPF group significantly promotes Bdnf protein expression while reducing the cleaved-caspase-3:caspase-3 ratio (Fig. 11*C*).

The above results indicate that propofol downregulates the expression of the Bndf gene and protein by activating Snhg1 through RARα while increasing the ratio of cleaved-caspase-3 to caspase-3.

Next, we assessed the viability of SK-N-SH cells induced by propofol and LDH release, oxidative stress, and cell apoptosis levels. The results showed that compared to the sh-NC + oe-NC + PPF group, the sh-RARa + oe-NC + PPF group and sh-Shng1 + oe-NC + PPF group significantly increased the viability of SK-N-SH cells, decreased the levels of LDH, ROS, and MDA while increasing SOD activity and reducing the apoptotic rate of the cells. Compared to the sh-RARa + oe-NC + PPF group, the sh-RARa + oe-Shng1 + PPF group significantly reduced the viability of SK-N-SH cells, while LDH levels, ROS levels, and MDA content significantly increased. At the same time, the rate of cell apoptosis also significantly increased. Compared to the sh-Shng1 + oe-NC + PPF group, the sh-Shng1 + oe-Bndf + PPF group significantly improved the viability of SK-N-SH cells and reduced LDH levels, ROS levels, and MDA content while increasing SOD activity and decreasing the apoptosis rate of cells (Fig. 11, *D*–*G*).

The above results indicate that downregulating RARα or Snhg1 significantly enhances the viability of SK-N-SH cells and reduces LDH levels, ROS levels, and MDA content while increasing SOD activity and decreasing cell apoptosis rate, whereas upregulating Snhg1 or Bdnf could reverse the above indicators. Thus, propofol induces toxicity in SK-N-SH cells by downregulating the Bndf gene through RARα-mediated transcriptional activation of Snhg1.

### Propofol Induces Neurotoxicity in Mice through RARα-Snhg1-Bdnf

Finally, we validated the regulatory network of RARα-Snhg1-Bdnf involved in propofol-induced neurotoxicity in mice. We used lentiviruses to knock down or overexpress RARα and Snhg1 separately to evaluate the effect of propofol on mouse neuroinjury. Cognitive levels were assessed through the Morris water maze behavioral test. The results showed that the escape latency significantly increased and the platform crossing times significantly decreased in the PPF group compared to the Saline group. Moreover, in comparison to the sh-NC + oe-NC + PPF group, the sh-RARa + oe-NC + PPF group and the sh-Snhg1 + oe-NC + PPF group exhibited a significant reduction in escape latency and a significant increase in platform crossing times.

**Figure 12.**
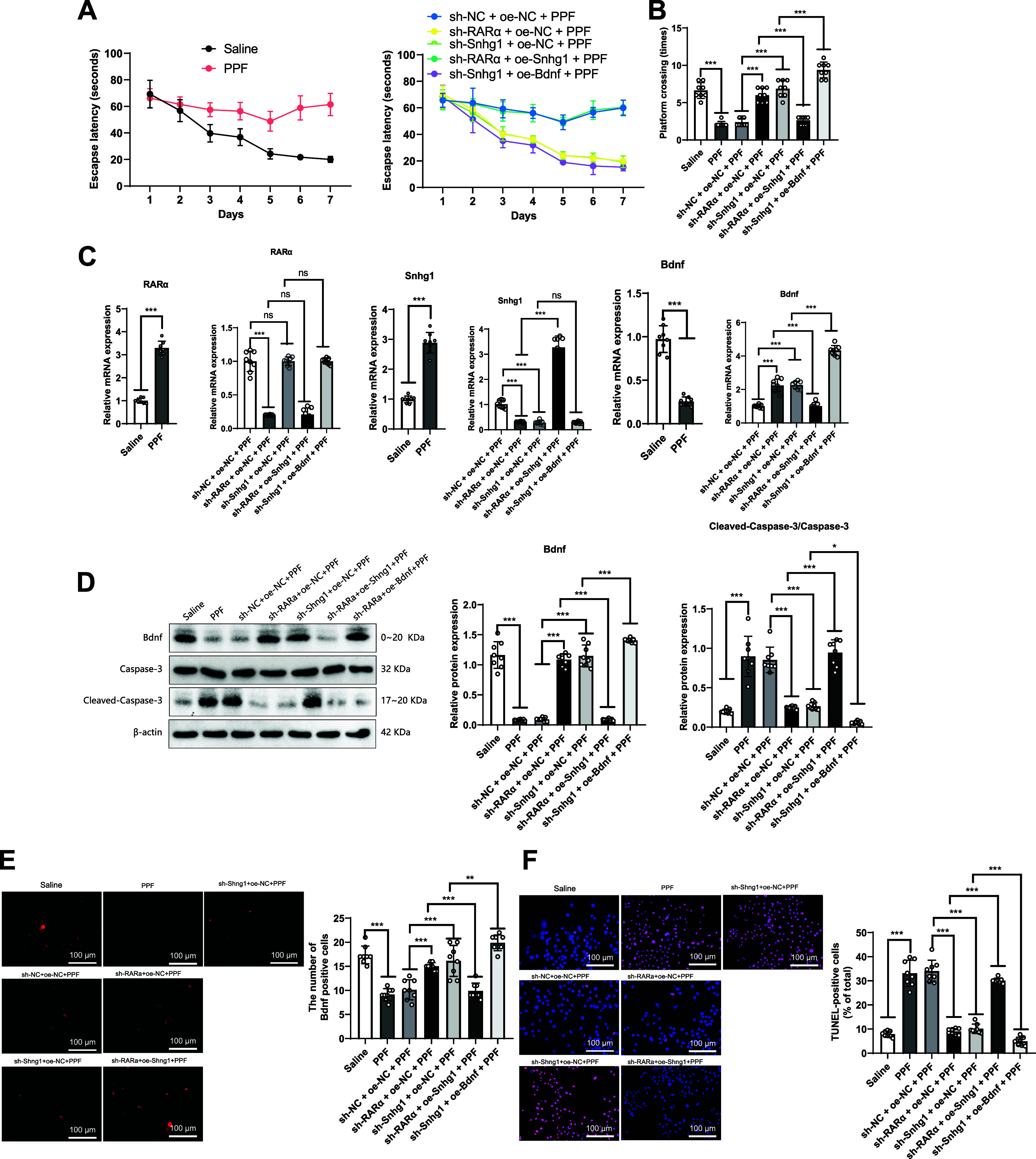
Propofol (PFF) affects neurotoxicity in mice through the retinoic acid receptor-α, small nucleolar RNA host gene 1, and brain-derived neurotrophic factor (RARα-Snhg1-Bdnf) pathway. Knockdown or overexpression of RARα and Snhg1 affects mice’s propofol-induced oxidative stress, apoptosis, and cognitive impairment. *A*: escape latency in the Morris water maze test. *B*: number of platform crossings in the Morris water maze test. *C*: gene expression of RARα, Snhg1, and Bdnf. *D*: protein expression of Bdnf and cleaved-caspase-3. *E*: immunofluorescence staining of Bdnf, with red dots representing labeled Bdnf protein. Scale bar = 100 μm. *F*: terminal deoxynucleotidyl transferase dUTP nick end labeling (TUNEL) staining, with blue dots representing cell nuclei and purple dots representing apoptotic cells. Scale bar = 100 μm; each group of mice, *n* = 8. Statistical analysis was performed using ANOVA for multiple group comparisons. *P < 0.05, in comparison between 2 groups; **P < 0.01; ***P < 0.001; ns, no significant difference; all experiments were repeated 5 times.

Additionally, the sh-RARa + oe-Snhg1 + PPF group demonstrated a significant increase in escape latency and a significant decrease in platform crossing times compared to the sh-RARa + oe-NC + PPF group. Furthermore, the sh-Snhg1 + oe-Bndf + PPF group showed a significant reduction in escape latency and increased platform crossing times compared to the sh-Snhg1 + oe-NC + PPF group (Fig. 12, *A* and *B*). The above results indicate that propofol induces behavioral changes in mice by regulating RARα-Snhg1-Bdnf, resulting in cognitive impairment.

We conducted experiments on mouse brain tissue obtained after the euthanasia of mice and detected the expression of RARα, Snhg1, and Bdnf-related genes. The results showed that compared to the Saline group, the PPF group exhibited significant upregulation of RARα and Snhg1 gene expression, while Bdnf gene expression was significantly downregulated. Compared to the sh-NC + oe-NC + PPF group, the gene expression of RARα and Snhg1 in the sh-RARa + oe-NC + PPF group was significantly downregulated, while the gene expression of Bdnf was significantly upregulated. The gene expression of Shng1 in the Sh-Shng1 + oe-NC + PPF group was significantly downregulated, while the gene expression of Bdnf was significantly upregulated. Compared to the sh-RARa + oe-NC + PPF group, the gene expression of Shng1 in the sh-RARa + oe-Shng1 + PPF group was significantly upregulated, while the gene expression of Bdnf was significantly downregulated. Compared to the sh-Shng1 + oe-NC + PPF group, the expression of the Bdnf gene was significantly upregulated in the sh-Shng1 + oe-Bndf + PPF group (Fig. 12*C*).

Western blot analyzed protein expression of Bdnf, cleaved-caspase-3, and caspase-3. The results showed that compared to the Saline group, the PPF group significantly suppressed Bdnf protein expression and increased the cleaved-caspase-3:caspase-3 ratio. Compared to the sh-NC + oe-NC + PPF group, the sh-RARa + oe-NC + PPF group and sh-Shng1 + oe-NC + PPF group significantly increased Bdnf protein expression and reduced the cleaved-caspase-3:caspase-3 ratio. Compared to the sh-RARa + oe-NC + PPF group, the sh-RARa + oe-Shng1 + PPF group significantly suppressed the expression of Bdnf protein and increased the ratio of cleaved-caspase-3 to caspase-3. Compared to the sh-Shng1 + oe-NC + PPF group, the sh-Shng1 + oe-Bndf + PPF group significantly promoted the expression of Bdnf protein and reduced the ratio of cleaved-caspase-3 to caspase-3 (Fig. 12*D*).

BDNF expression was detected by immunofluorescence staining, while neuronal apoptosis was assessed in mouse brain tissue through TUNEL staining. The results revealed that compared to the Saline group, the PPF group exhibited a significant decrease in BDNF protein expression and a significant increase in apoptotic cells. Furthermore, compared to the sh-NC + oe-NC + PPF group, both the sh-RARa + oe-NC + PPF group and the sh-Snhg1 + oe-NC + PPF group showed a significant increase in BDNF levels and a significant reduction in apoptotic cells. In contrast, the sh-RARa + oe-Snhg1 + PPF group displayed a significant decrease in BDNF expression and an increase in apoptotic cells compared to the sh-RARa + oe-NC + PPF group. Similarly, compared to the sh-Snhg1 + oe-NC + PPF group, the sh-Snhg1 + oe-Bdnf + PPF group exhibited an increase in BDNF expression and a significant decrease in apoptotic cells (Fig. 12, *E* and *F*). In conclusion, the above results indicate propofol induces neurotoxicity in mouse brain neurons by regulating RARα-Snhg1-Bdnf.

## DISCUSSION

Propofol has been widely used for induction and maintenance of general anesthesia in clinical settings, as well as providing procedural sedation and coma. It peaks at 100 s after injection of 2.5 mg/kg and lasts 5–10 min. It decreases respiratory drive, weakens protective airway reflexes, and can lower upper airway muscle tone, leading to airway obstruction (42, 43). This study explains how propofol regulates the induction of neurotoxicity in brain neurons through the RARα-Snhg1-Bdnf pathway. These findings have significant implications for clinical practice (44). Clinicians can make more informed decisions regarding propofol usage by understanding its molecular mechanism comprehensively, allowing for a more precise assessment of its risk (45). The experimental results have also generated novel ideas and avenues for identifying potential treatment or prevention strategies in clinical practice (46). Not only can this reduce the neurotoxic risk caused by propofol, but it may also help scientists in developing safer alternatives to anesthetic drugs (47). In general, this study offers crucial theoretical support to enhance the safety and efficacy of propofol in clinical applications (48).

This study explored the molecular mechanisms underlying propofol-induced neurotoxicity in the brain and evaluated the efficacy of both bioinformatics and experimental methods (49). When choosing relevant chip data from the GEO database, we prioritize the accuracy and reliability of the data to ensure high-quality research findings (50). Furthermore, we have extensively utilized diverse bioinformatics tools to validate our research findings and strengthen the study’s credibility (51). Simultaneously, we extensively discussed the strengths and limitations of the experimental design to comprehensively assess the scientific merit and feasibility of the study (52).

This study extensively explores the molecular mechanisms that underlie the neurotoxic effects of propofol on the brain (53). Our study revealed that propofol hinders the expression of Bdnf by promoting the binding between RARα and the Snhg1 promoter region, resulting in neurotoxicity (54). This discovery is highly significant as it represents a breakthrough in understanding drug toxicity (55). Simultaneously, we explored additional potential mechanisms of toxicity that enhance our comprehensive understanding of the occurrence of neurotoxicity (56).

The experimental results of this study elucidated the molecular mechanism by which propofol regulates neurotoxicity in brain neurons via the RARα-Snhg1-Bdnf regulatory network (53). This discovery holds significant implications for comprehending the mechanisms behind drug-induced neural damage, emphasizing the pivotal role of this regulatory network in neurotoxic processes (57). Moreover, the experimental findings have also stimulated significant contemplation regarding clinical applications, particularly in enhancing the monitoring and management of propofol usage to mitigate potential risks patients encounter during clinical practice (58).

Our study implicates propofol in inhibiting Bdnf expression and inducing neurotoxicity in the brain by promoting RARα binding to the Snhg1 promoter region (54). This discovery offers insight into neurotoxic mechanisms, providing essential clues for future research on neuroprotection and clinical treatment (59). However, it is essential to acknowledge the limitations of this study, particularly the use of cell and animal models. Therefore, further validation is necessary before applying these findings to clinical practice (60). Furthermore, additional unidentified regulatory networks and mechanisms might warrant further investigation in the future (61). However, the findings of this study are potentially significant for research on neurotoxicity and the development of neuroprotective strategies (62).

**Figure 13.**
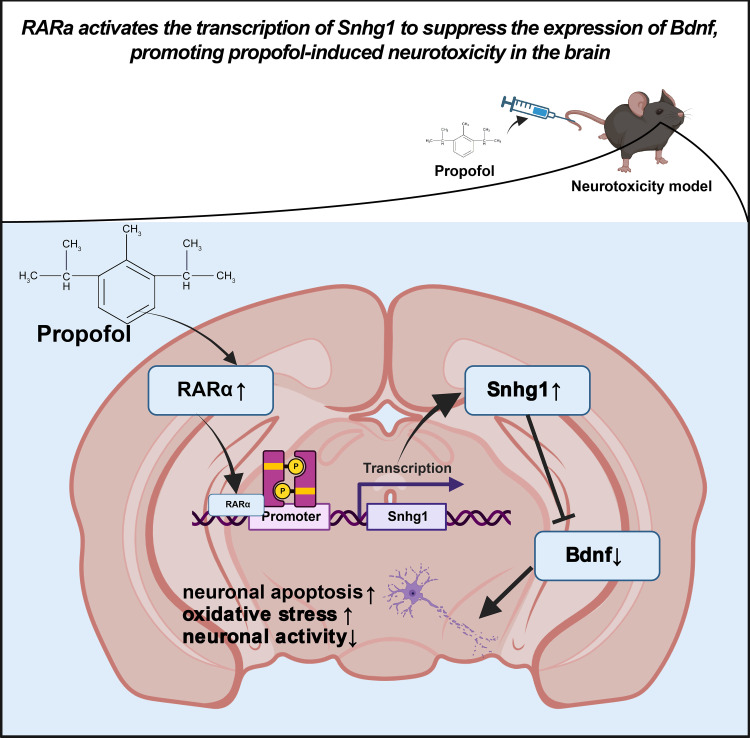
Schematic diagram of the molecular mechanisms by which retinoic acid receptor-α (RARα) regulates the expression of small nucleolar RNA host gene 1 (Snhg1) and influences propofol-induced neurotoxicity in the brain. Bdnf, brain-derived neurotrophic factor.

In summary, we have clarified the molecular mechanism by which propofol inhibits the expression of Bdnf (Fig. 13). This is achieved by promoting the binding of RARα to the promoter region of Snhg1. This discovery provides significant insights into understanding the neurotoxicity of propofol and offers crucial guidance for future clinical practice and drug treatment strategies. Nonetheless, this study still has limitations, including the presence of unidentified regulatory networks and mechanisms that necessitate future investigations for clarification.

## ETHICS APPROVALS

All animal experiments strictly adhered to the *Guidelines on the Care and Use of Laboratory Animals* set by the National Institute of Health and have been approved by our institutional Animal Ethics Committee of Air Force Medical Center (2023-180-S01).

## DATA AVAILABILITY

The datasets generated and/or analyzed during the current study are available from the corresponding author upon reasonable request.

## GRANTS

This study was supported by Air Force Medical Center Youth Talent Fund (2022YXQN013).

## DISCLOSURES

No conflicts of interest, financial or otherwise, are declared by the authors.

## AUTHOR CONTRIBUTIONS

Y.X. and T.T. conceived and designed research; Y.X. performed experiments; Y.X. analyzed data; X.X. and T.T. interpreted results of experiments; X.X. and T.T. prepared figures; Y.X. and T.T. drafted manuscript; X.X. edited and revised manuscript; Y.X., X.X., and T.T. approved final version of manuscript.
